# Neoadjuvant chemotherapy alters peripheral and tumour‐infiltrating immune cells in breast cancer revealed by single‐cell RNA sequencing

**DOI:** 10.1002/ctm2.621

**Published:** 2021-12-17

**Authors:** Hanlin Zhou, Guibo Li, Jianhua Yin, Ting Wang, Hong Hu, Tong Li, Qing Zhou, Jintao Hu, Lei Wang, Shichen Dong, Chen Wei, Cuijuan Zhang, Ye Lu, Wenbin Zhou, Chen Huang, Huanming Yang, Dongxian Zhou, Ling Wang, Kui Wu

**Affiliations:** ^1^ BGI‐Shenzhen Shenzhen China; ^2^ BGI College & Henan Institute of Medical and Pharmaceutical Science Zhengzhou University Zhengzhou China; ^3^ BGI‐Henan BGI‐Shenzhen Xinxiang China; ^4^ Guangdong Provincial Key Laboratory of Human Disease Genomics Shenzhen Key Laboratory of Genomics BGI‐Shenzhen Shenzhen China; ^5^ Laboratory of Genomics and Molecular Biomedicine Department of Biology University of Copenhagen Copenhagen Denmark; ^6^ Department of Vascular and Endocrine Surgery Xijing Hospital Fourth Military Medical University Xi'an China; ^7^ Department of Breast Surgery The Second Affiliated Hospital of Jinan University Shenzhen People's Hospital Shenzhen China; ^8^ Department of Pathology The Second Affiliated Hospital of Jinan University Shenzhen People's Hospital Shenzhen China; ^9^ College of Life Sciences University of Chinese Academy of Sciences Beijing China; ^10^ Omics team James D. Watson Institute of Genome Sciences Hangzhou China

Dear Editor,

Neoadjuvant chemotherapy (NAC) is one of the standard preoperative therapeutic approaches for breast cancer to downsize tumours, but its clinical efficacy is diverse among patients.^1^ To understand the causes of disparity in patient outcomes, it is critical to unveil how NAC remodels circulatory immune cells and tumour microenvironment (TME). In this study, we performed single‐cell RNA sequencing of 132 823 cells isolated from primary breast tumours with different molecular subtypes, peripheral blood mononuclear cells (PBMCs) collected at three timepoints during patients undergoing NAC and post‐NAC tumours respectively (Figure 1A, Table S1).

**FIGURE 1:**
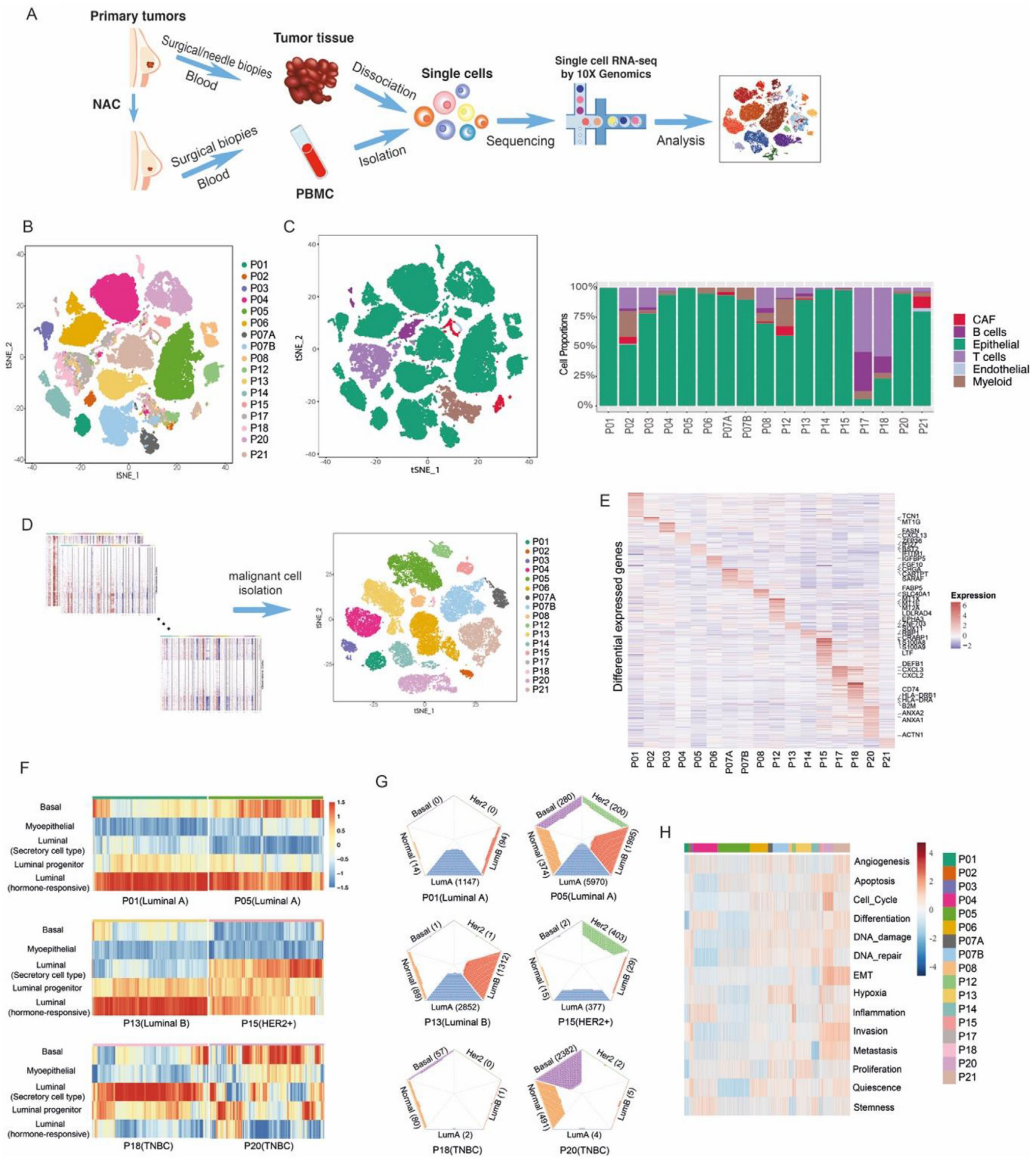
Heterogeneity of malignant cells in 17 primary breast cancer tumours. (A) Work flow of this study. (B) t‐SNE plot of 57 777 cells from 17 primary breast cancer tumours. The cells are coloured by sample origin. (C) t‐SNE plot as in (B), coloured by inferred cell types, and proportional distribution of different cell types across 17 samples. (D) t‐SNE plot of all tumour cells isolated by inferCNV, coloured by sample origins. (E) Differentially expressed genes of tumour cells from 17 patients. (F) Heatmap of the corresponding expression of normal epithelial gene signatures in each epithelial cell from 6 patients with distinct molecular subtypes. (G) Breast cancer molecular subtypes of single cells predicted by the PAM50 classifier. (H) Heatmap depicting the expression of gene signatures from the CancerSEA database in each tumour cell from 17 primary breast cancer tumours

First, we dissected the TME of 17 primary breast carcinomas by separating total cell populations into tumour cells, stromal cells and tumour‐infiltrating immune cells. Independent analysis on the amount and gene features of each cell type was then conducted. Immune cells and stromal cells from different samples can be clustered together based on their cell types, while epithelial cells originating from different tumours were separately distributed on the t‐SNE plot, suggesting a high degree of intertumour heterogeneity (Figures 1B, C and S1A).

We isolated malignant cells from epithelial cells through assessing the copy number variation (CNV) inference (Figures 1D and S1B). Most patients exclusively expressed higher levels of one or more oncogenesis‐associated genes (Figure 1E). We then compared the epithelial features of the malignant cells with published normal breast epithelia data. Our results showed that malignant cells from a single tumour could harbour the gene expression profiles derived from multiple subsets of normal epithelial cells (Figures 1F and S2). When analysed by the PAM50 classifier, tumour cells from an individual patient could be classified into multiple molecular subtypes, which demonstrated the presence of heterogeneity within a breast tumour (Figure 1G and S3). In the subsequent analysis of 14 functional states of malignant cells, we observed that epithelial–mesenchymal transition (EMT), invasion and metastasis signatures were enriched in samples from patients with triple‐negative breast cancer (TNBC) (Figure 1H), which is consistent with previous findings indicating a more aggressive subtype of breast cancer.^2^

We then analysed the immune compartment and identified subclusters of T cells, B cells and macrophages. The results were consistent with those of previous studies, in which cytotoxic T cells, regulatory T cells, exhausted T cells, germinal centre (GC) B cells, plasma B cells, dendritic cells (DCs) and plasmacytoid‐DCs were identified according to the expression of relevant marker genes (Figure 2A–D).^3^ Our cell–cell communication analysis revealed that T cells had more positive interaction with tumour cells (Figure 2E, F). Immunosuppressive interactions between tumour cells and T cells were identified including the ligand‐receptor pairs NECTIN1–CD96 and NECTIN2–TIGIT (Figure 2G). This finding therefore suggested a plausible approach for breast carcinomas to escape from immune supervision and that TIGIT is a potential immune checkpoint of CD8+ T cells in breast cancer.

**FIGURE 2:**
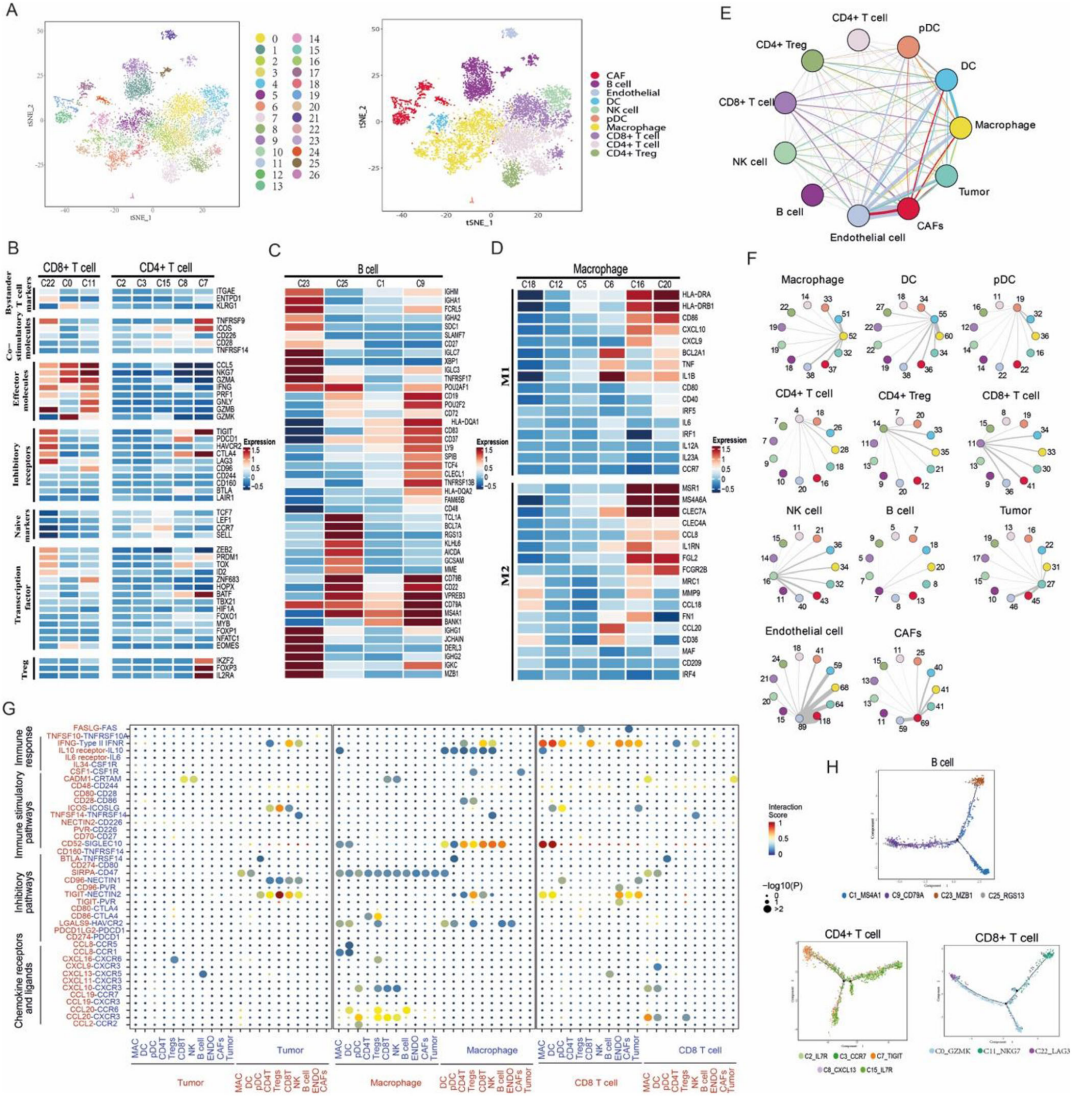
The immune cell profiles in the breast cancer TME. (A) t‐SNE plot of the cells excluding epithelial cells, coloured by the clusters (left) or the annotated cell types (right). (B) The gene expression of T cell functional states in each cluster of CD8+ T cells and CD4+ T cells was demonstrated. (C) Heatmap as in (B), indicating the gene expression of B cell functional states in B cell subpopulations. (D) The expression of M1 and M2 gene signatures in each cluster of macrophages showed by the heatmap. (E) Density of cell–cell interactions between each two cell types. Each line starting from a certain cell population sharing the same colour represents the ligands expressed by the cell type. The other end of the line connects the cells expressing corresponding receptors. The thickness of each line proportionally indicates the number of ligand‐receptor pairs between two cell types. (F) Detailed view of the numbers of ligand‐receptor pairs between each two cell types. (G) The dot plot of the selected ligand and receptor interactions in tumour cells, macrophages and CD8+ T cells. The *p* value is shown by the circle size. The interaction score is labelled with colour. MAC, macrophage; DC, dendritic cell; Tregs, regulatory T cells; NK, natural killer cells; ENDO, endothelial cells; CAFs, cancer‐associated fibroblasts; pDC, plasmacytoid dendritic cells. (H) The pseudotime trajectories derived from CD8+ T cells, CD4+ T cells and B cells. Each dot corresponds to one single cell, coloured by the relative clusters

We next analysed the TME and PBMCs of patients treated with NAC (Figures 3A, B and 4A, B). In paired tumours (pre‐ and post‐treatment) from patient BC06, the malignant cells were isolated by inferCNV. We observed multiple enriched pathways associated with malignancy including angiogenesis, EMT and metastasis in post‐treatment tumours (Figure 3E). Through PAM50 analysis, the compositions of tumour epithelial phenotypes were also found to be altered by NAC (Figure 3F). The fractions of T cells and B cells were decreased in the post‐treatment tumour compared to the pre‐treatment tumour of patient BC18 (Figure 3C) with diminished copy number aberrances (Figure 3D). The IFNα and IFNγ signalling pathways were consistently downregulated in immune cell populations of the post‐treatment tumour (Figure 3H). In the analysis of paired tumours from patients BC06 and BC18, we demonstrated that NAC had not only reshaped the heterogeneity and functional features of tumour cells but also changed the proportions of tumour‐infiltrating lymphocytes and inhibited the immune activation.

**FIGURE 3:**
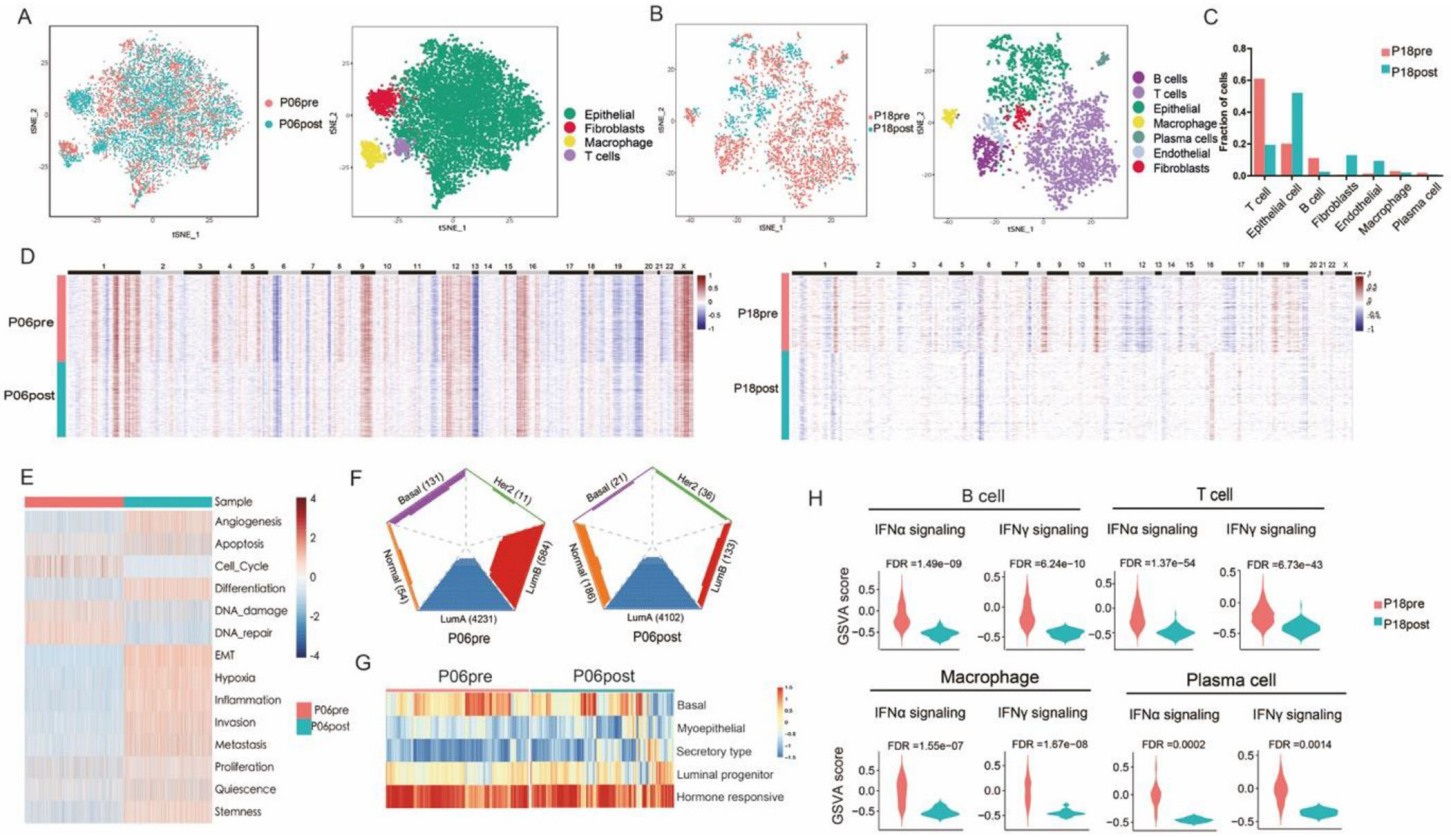
The TME alterations of paired P06 and P18 pre‐ and post‐treatment tumours. (A) t‐SNE plot of single cells of P06 pre‐ and post‐treatment tumours, coloured by cell types. (B) t‐SNE plot of single cells of P18 pre‐ and post‐treatment tumours, coloured by the annotated cell types. (C) The fraction of each cell type in pre‐ and post‐treatment tumours of patient BC18. (D) The CNV alteration of epithelial cells isolated from P06 and P18 pre‐ and post‐ treatment tumours. (E) Heatmap of gene signatures related to cancer cell functional status of malignant cells from P06 pre‐ and post‐treatment tumours. (F) Tumour epithelial gene features of malignant cells from P06 pre‐ and post‐treatment tumours using PAM50 classifier. (G) Normal epithelial gene features shared by malignant cells from P06 pre‐ and post‐treatment tumours. (H) The altered pathways identified by GSVA in major immune cells from P18 pre‐ and post‐treatment tumours. FDR < 0.01

**FIGURE 4:**
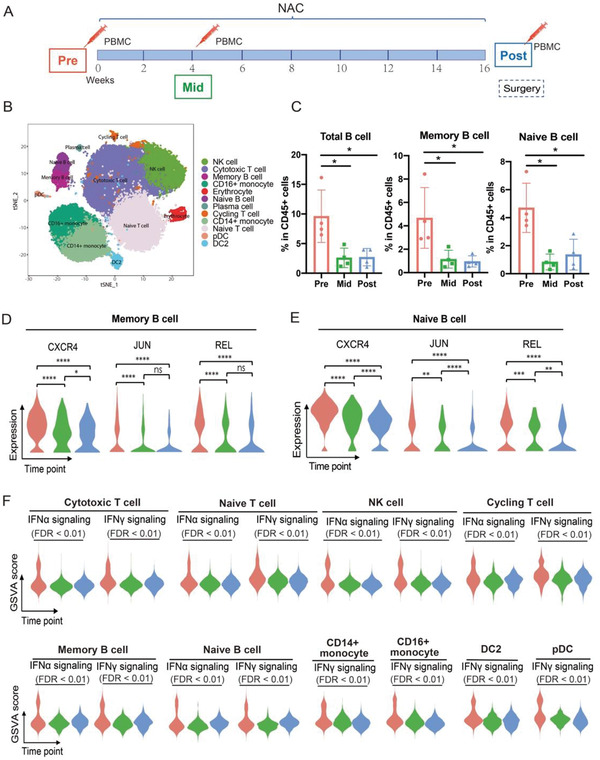
Molecular characterisation of immune cells in PBMCs from four patients during NAC. (A) Scheme of PBMC samples collected from patients undergoing NAC. (B) t‐SNE plot of PBMC single cells collected from pre‐, mid‐ and post‐NAC stages. The cells are coloured by the annotated cell type. (C) Fraction of B cell subpopulations from pre‐, mid‐ and post‐NAC samples. Two‐tailed Mann–Whitney U analysis, *p < .05. Data was shown with mean ±SD (n = 4 per group). (D) Violin plots of differentially expressed genes in memory B cells across all timepoints. Wilcox rank sum test, *p < .05, ****p < .0001, ns indicates not significant. (E) Violin plots of differentially expressed genes in naïve B cells across all timepoints. Wilcox rank sum test, *p < .05, **p < .01, ***p < .001, ****p < .0001. (F) Violin plots representing the differentially enriched gene sets discovered by GSVA in each cell type across all timepoints, FDR < 0.01

Consistently, the fraction of B cells was significantly reduced during and after NAC in PBMCs, suggesting that the chemotherapy could affect the humoral immune response (Figure 4A–C). CXCR4, which is involved in maintaining the B cell population and function,^4^ was significantly downregulated by NAC as well (Figure 4D, E). The declined levels of STAT1, JUN and NFKBIZ in B cells, cytotoxic T cells,

NK cells and DC2 cells respectively from pre‐treatment stage to mid‐ and post‐treatment stages indicated that the activation, proliferation and differentiation of these cells could be inhibited by NAC (Figures 4D, E and S4A, B, C).^5^, ^6^ We also noticed a slight increase in LAG3 and TIGIT mRNA levels in cytotoxic T cells after NAC (Figure S4A), which implies the occurrence of immune exhaustion induced by NAC.^7^ The GSVA results showed that the IFNα and IFNγ responses were downregulated in most immune cells after NAC in blood (Figure 4F). Moreover, the pathways which were responsible for transducing type I IFN signals, such as the JAK‐STAT1 pathway, were also found to be decreased in T cells, NK cells and myeloid cells (Figure S4D).^8^ Collectively, these results suggested that the circulatory and local immune cells could be inhibited during or after NAC. The results were opposite to some previous findings that immune activation were commonly observed after NAC.^9^ However, a recent study revealed that the immune suppression in TME after NAC was linked to patients' resistance to the therapy and indicated poor prognosis, whereas patients with immune restores could benefit.^10^

In summary, our work provides a comprehensive single‐cell transcriptome atlas of the microenvironment in breast tumour including malignant, stromal and immune cells. These results uncover detailed intratumour heterogeneity, immune diversity and the complex communication network of cancer cells and other components. In addition, the single‐cell analysis on paired breast tumour samples as well as PBMCs collected during NAC proved that the therapy could reshape the systemic and local immune microenvironments, and the tumours in our study may enter the equilibrium and escape phases after NAC through decreased IFNα and IFNγ responses. In near future, prospective studies with more paired tumour biopsies from patients with pre‐ and post‐treatment would be conducted to further confirm our findings and perform extensive in‐depth investigation.

## Supporting information

Supplement information
