# Supplementary material for: Neoadjuvant chemotherapy alters peripheral and tumour‐infiltrating immune cells in breast cancer revealed by single‐cell RNA sequencing
Source: Clin Transl Med. 2021 Dec 17;11(12):e621. doi: 10.1002/ctm2.621 (PMC8679836; doi:10.1002/ctm2.621)
Supplement: Supplementary file 1 — Supplement information [file CTM2-11-e621-s001.docx]

Supplemental materials

**Materials and methods**

*Patients and human specimens*

Eighteen patients were enrolled in this study and assigned into four molecular subtypes in the clinic (Table S1): luminal A (BC01-BC10), luminal B (BC12, BC13 and BC14), HER-2 (BC15) and TNBC (BC17, BC18, BC20 and BC21). Seventeen primary tumor samples were collected from these patients including biopsies and surgical sections (Table S2). Samples P07A and P07B were from different sites of the tumor of patient BC07. P06post and P18post were from postoperative residual tumors of patients BC06 and BC18 who underwent NAC respectively. PBMCs were obtained from four patients (BC06, BC09, BC10 and BC18) in the three stages during NAC (pre-, mid-, and post-treatment). This study was approved by the Institutional Review Board of BGI (BGI-IRB, N0: BGI-IRB 17090) and Shenzhen People’s Hospital (Second Clinical Medical College of Jinan University). Written informed consent was acquired from the subjects or their authorized representatives.

*Tissue procession and single cell collection*

The tumor tissues were minced into tiny cubes <1 mm^3^ on ice and transferred into a 1.5 ml tube contenting 20 U/ml collagenase III (Invitrogen), 3 U/ml hyaluronidase (Invitrogen), 1 U/ml DNase I (Invitrogen) and 1× Hank's balanced salt solution (HBSS, Invitrogen). The tumor pieces were digested in this digestion medium for 60 min at 37°C. Then, the tumor cells were filtered by a 40 μm cell strainer (BD) and centrifuged at 300 $\times$g and 4°C for 5 min. After centrifugation, the supernatant was discarded, and the cell pellet was resuspended in PBS with 0.02% BSA (Sigma). PBMCs were isolated from whole blood using Ficoll-Paque (Eppendorf) according to the manufactured protocol. The cells were washed with PBS and filtered through 40 μm cell strainer. After centrifugation, the cell pellet was resuspended in PBS with 0.02% BSA.

*Single cell RNA sequencing and data processing*

Cells were concentrated to 700-1000 cells/μl and run on a Chromium Single-Cell Instrument (10X Genomics) to generate single-cell gel bead-in-emulsions (GEMs). The Chromium Single Cell 3' Reagent Kit V1 or V2 (10X Genomics) was applied for RNA reverse transcription, cDNA recovery, amplification and library construction according to the manufacturer's protocol. In the sequence library constructed with the GemCode Single-Cell 3' Library V1 Kit, the first read consisted of 14-bp cell barcodes and 10-bp unique molecular identifiers (UMIs), and the second read had 100-bp 3' transcripts and 8-bp sample indexes. Another library was constructed with the GemCode Single-Cell 3' Library V2 Kit, in which the first read consisted of 16-bp cell barcodes and 10-bp UMIs, and the second read consisted of 100-bp 3' transcripts and 8-bp sample indexes. The sequencing libraries were loaded onto the BGISEQ-500 platform for paired-end read sequencing. Raw FASTQ files were processed with CellRanger v1.2 and v2.0 (10X Genomics). The sequence reads were aligned to the GRCh38 reference transcriptome using STAR and a filtered UMI expression profile for each cell was generated (*1, 2*).

*Cell filtration and clustering*

Data from primary tumors were merged into one gene cell barcode matrix and quality filtering was performed. Cells with 400 to 8000 expressed genes and with less than 20% mitochondrial UMI counts were retained. Genes present in at least 10 cells in one sample were kept for further analysis. In total, 57,777 single cells and 23,440 genes passed the QC criteria and were analyzed by the Seurat R package (version 3.0, https://satijalab.org/seurat/). Highly variable genes were calculated with the Find Variable Genes method in Seurat. The genes with a mean expression value between 0.0125 and 4 and a dispersion value greater than 0.5 were selected to perform principal component analysis (PCA) for dimensionality reduction on a log-normalized data matrix. The cells were clustered into 59 groups by the FindClusters method using the top 40 principal components (PCs).

*CNV inference and malignant cell identification*

The InferCNV package (version 0.1) was applied to evaluate the CNVs of each cell (*3*). PBMC samples from a healthy donor served as the reference. The data of the reference and our samples were transformed with log2 (TPM+1) to calculate the CNV scores by InferCNV using default parameters. We performed k-means clustering (k=2) with the CNV scores of these samples. The epithelial cells from tumor samples that were predominantly clustered with cells from normal controls were classified as nonmalignant, while the cells that harbored copy number alterations and were clustered separately were defined as malignant.

*Non-epithelial cell annotation*

All non-epithelial cells were clustered into 27 groups by the Seurat FindClusters method using the top 20 PCs. Cluster-specific genes were used to identify the cell types. T cells: CD3D, CD2, CD3E and CD3G; B cells: CD19, CD79A and CD79B; NK cells: NKG7 and GNLY; macrophages: CD163, CSF1R and CD14; cDCs: CLEC10A, CD1C and CD1E; pDCs: LILRA4 and IL3RA; CAFs: COL1A1, COL1A2 and COL3A1; endothelial cells: VWF, PECAM1 and CLDN5.

*Molecular feature prediction of malignant cells*

The normal epithelial feature scores for each malignant cell were calculated using the function AddModuleScore in Seurat. The applied five epithelial signatures are from published single-cell data of normal breast epithelia (*4*).

We utilized the PAM50 classifier to predict the intrinsic molecular subtypes (normal-like, basal, HER2, luminal B, and luminal A) of each tumor cell using the genefu R package with the transformed log2 (TPM+1) data (*5, 6*). Single cells with high prediction confidence ($\geq$0.7) were set as a defined breast cancer subtype.

*Constructing Single-Cell Trajectories*

Trajectory analysis was conducted with Monocle2 (version 2.6.4, http://cole-trapnell-lab.github.io/monocle-release/). Differentially expressed genes (DEGs) were identified by differential gene expression analysis using the differentialGeneTest function in Monocle2. The top 1,000 significant DEGs with q values < 0.01 were used to order cells. After dimensionality reduction and cell ordering, trajectories were established and visualized by the plot_cell_trajectory function in Monocle2 according to pseudotime.

*Cell-cell communication analysis*

Cell-cell communication analysis was performed with the CellPhoneDB python package (version 1.1.0) in which a public repository of ligands, receptors and their interactions is available (*7*). The normalized cell matrix derived from the Seurat normalization object was input into CellPhoneDB. Abundant ligand-receptor interactions between two cell types were discovered according to the expression of a receptor by one cell type and the expression of the corresponding ligand by the other cell type. Pairwise comparisons were performed between the included cell types. The p value corresponding to the cell type specificity of the ligand-receptor complex was obtained through the CellPhoneDB algorithm.

*Integration analysis of paired tumor tissues and PBMCs*

The Seurat alignment method CCA was applied for data integration in the analysis of paired tumor samples (*8*). We calculated the shared subspace on the basis of the aligned canonical correlation components. A new low-dimensional reduction result was generated for further analysis including cell clustering by the FindClusters method and visualization by t-SNE analysis in the Seurat R package (version 2.3.4). The Harmony algorithm was used to integrate the data of 12 PBMC samples (*9*). The top 30 PCs were chosen to perform the data integration by calling the RunHarmony function in Harmony, and then the data were processed in Seurat as mentioned above.

*Differential gene expression analysis*

We conducted differential gene expression analysis by the FindMarkers function with the Wilcox rank sum test algorithm in Seurat. Genes with a fold change of no less than 2 and an adjusted-p value of less than 0.05 were retained. A heatmap of these genes was constructed by the R packages ggplot2 (version 3.2.0, <https://ggplot2.tidyverse.org/>) and ComplexHeatmap (version 1.12.0) (*10*).

*Gene set variation analysis (GSVA)*

GSVA was applied to identify the molecular phenotypes of single cells using the log2 (TPM+1) data (*11*). First, the GSVA scores for 5,501 curated gene sets (MSigDB version 7) were calculated for each cell (*12*). For each cell type from the paired samples, the GSVA scores of the total cells were compared by using the R package limma (*13*). Differentially enriched gene sets were regarded as significant with FDR-adjusted p value < 0.05.

The GSVA scores for the tumor cells were calculated by the 14 functional gene signatures in the CancerSEA database. Then comparisons between the GSVA scores of the 14 gene signatures were performed with the rcorr function in the R package Hmisc (version 4.3.0. <https://cran.r-project.org/web/packages/Hmisc/index.html>). Then, a heatmap of the correlations among the 14 gene signatures was constructed by the R packages ggplot2 (version 3.2.0) and ComplexHeatmap (version 1.12.0).

*Statistical analysis*

Data analysis was performed with R studio (version 1.3) or GraphPad Prism 8.0. A two-tailed Mann-Whitney *U* test or Wilcox rank sum test were used to compare the values of every two groups. In GSVA, FDR-adjusted p value is calculated by "limma" using Benjamini-Hochberg (BH) correction. Differences with p values < 0.05 or FDR < 0.05 were described as statistically significant.

**Acknowledgements**

**Fundings**: This work was supported by the National Key Research and Development Program of China (No. 2017YFC1309100); the Science and Technology Planning Project of Guangdong Province (2017B020227012); the Science, Technology and Innovation Commission of Shenzhen Municipality (Grant No. JCYJ20170412153155228); the Shenzhen Key Laboratory of Single-Cell Omics (ZDSYS20190902093613831) and the National Natural Science Foundation of China (No. 81672593, 81272899, 31970857); the Major Program for Natural Science Foundation of Shaanxi Province (No.2021JZ-29). This research was supported by the Guangdong Enterprise Key Laboratory of Human Disease Genomics (2020B1212070028).

**Author contributions**: GBL, KW, JHY, DXZ and LW designed and directed the study. HLZ, SCD, CW and YL performed the experiments and analyzed single cell RNA-Seq. TW, HH, JTH, WBZ and CH coordinated the samples. QZ, LW, and HMY contributed to scientific discussions for designing the study. HLZ, GBL, JHY, TW, HH and TL wrote the manuscript. All authors read and approved the final manuscript.

**Competing interests**: The authors declare that they have no competing interests.

**Data and material availability**: All raw sequencing data has been submitted to the CNGB Nucleotide Sequence Archive (CNSA) (*14*) of the China National GeneBank DataBase (CNGBdb) (*15*) with accession no. CNP0001356.

**Table S1. Clinical and histological profiles of the breast cancer specimens.**

| Patient index | Age | | Pathologic stage | Molecular subtype | ER | PR | HER-2 (IHC) | HER-2 (FISH) | ki-67 | NAC |
| --- | --- | --- | --- | --- | --- | --- | --- | --- | --- | --- |
| BC01 | 36 | T1N0M0 (IA) | | Luminal A | 99% | 99% | + | - | 15% |  |
| BC02 | 62 | T1N0M0 (IA) | | Luminal A | 90% | 90% | - | - | 10% |  |
| BC03 | 35 | T2N2M0 (IIIA) | | Luminal A | 90% | 80% | + | - | 10% |  |
| BC04 | 49 | TisN0M0 (Tis) | | Luminal A | 90% | 90% | ++ | - | 5% |  |
| BC05 | 63 | T2N0M0 (IIA) | | Luminal A | 90% | 90% | + | - | 20% |  |
| BC06 | 60 | T2N2M0 (IIIA) | | Luminal A | 95% | 95% | - | - | 15% | ECT |
| BC07 | 21 | T2N0M0 (IIA) | | Luminal A | 60% | 30% | ++ | Negative | 15% |  |
| BC08 | 62 | T1N0M0 (IA) | | Luminal A | 90% | 85% | ++ | Negative | 25% |  |
| BC09 | 63 | T2N0M0 (IIA) | | Luminal A | 90% | 90% | + | - | 10% | ECT |
| BC10 | 52 | T1N1M1 (IV) | | Luminal A | 75% | 1% | ++ | Negative | 5% | ECT |
| BC12 | 52 | T2N0M0 (IIA) | | Luminal B | 80% | 5% | ++ | - | 25% |  |
| BC13 | 57 | T1N0M0 (IA) | | Luminal B | 90% | 5% | ++ | Negative | 25% |  |
| BC14 | 59 | T1N0M0(IA) | | Luminal B | 50% | - | +++ | Negative | 40% |  |
| BC15 | 63 | T2N0M0 (IIA) | | HER-2+ | 10% | - | +++ | Positive | 15% |  |
| BC17 | 48 | T1N0M0 (IA) | | TNBC | - | - | - | - | 10% |  |
| BC18 | 43 | T1N0M0 (IA) | | TNBC | - | - | - | - | 70% | ECT |
| BC20 | 45 | T1N0M0(IA) | | TNBC | - | - | + | - | 90% |  |
| BC21 | 59 | T1N1M1 (IV) | | TNBC | - | - | ++ | - | 45% |  |

TNBC, triple-negative breast cancer; ER, estrogen receptor; HER2, human epidermal growth factor receptor 2; PR, progesterone receptor; FISH, fluorescence in situ hybridization; Y, neoadjuvant chemotherapy; N, without neoadjuvant chemotherapy; ECT, Epirubcin, Cyclophosphamide and Taxol.

**Table S2. Summary of samples and patient metadata for scRNA-seq.**

| Patient index | Primary tumor ID | Residual Tumor ID after NAC | PBMC ID before NAC | PBMC ID in NAC | PBMC ID after NAC |
| --- | --- | --- | --- | --- | --- |
| BC01 | P01 | - | - | - | - |
| BC02 | P02 | - | - | - | - |
| BC03 | P03 | - | - | - | - |
| BC04 | P04 | - | - | - | - |
| BC05 | P05 | - | - | - | - |
| BC06 | P06 | P06post | PB06pre | PB06mid | PB06post |
| BC07 | P07A  P07B | -  - | -  - | -  - | -  - |
| BC07 |  |  |  |  |  |
| BC08 | P08 | - | - | - | - |
| BC09 | - | - | PB09pre | PB09mid | PB09post |
| BC10 | - | - | PB10pre | PB10mid | PB10post |
| BC12 | P12 | - | - | - | - |
| BC13 | P13 | - | - | - | - |
| BC14 | P14 | - | - | - | - |
| BC15 | P15 | - | - | - | - |
| BC17 | P17 | - | - | - | - |
| BC18 | P18 | P18post | PB18pre | PB18mid | PB18post |
| BC20 | P20 | - | - | - | - |
| BC21 | P21 | - | - | - | - |

**
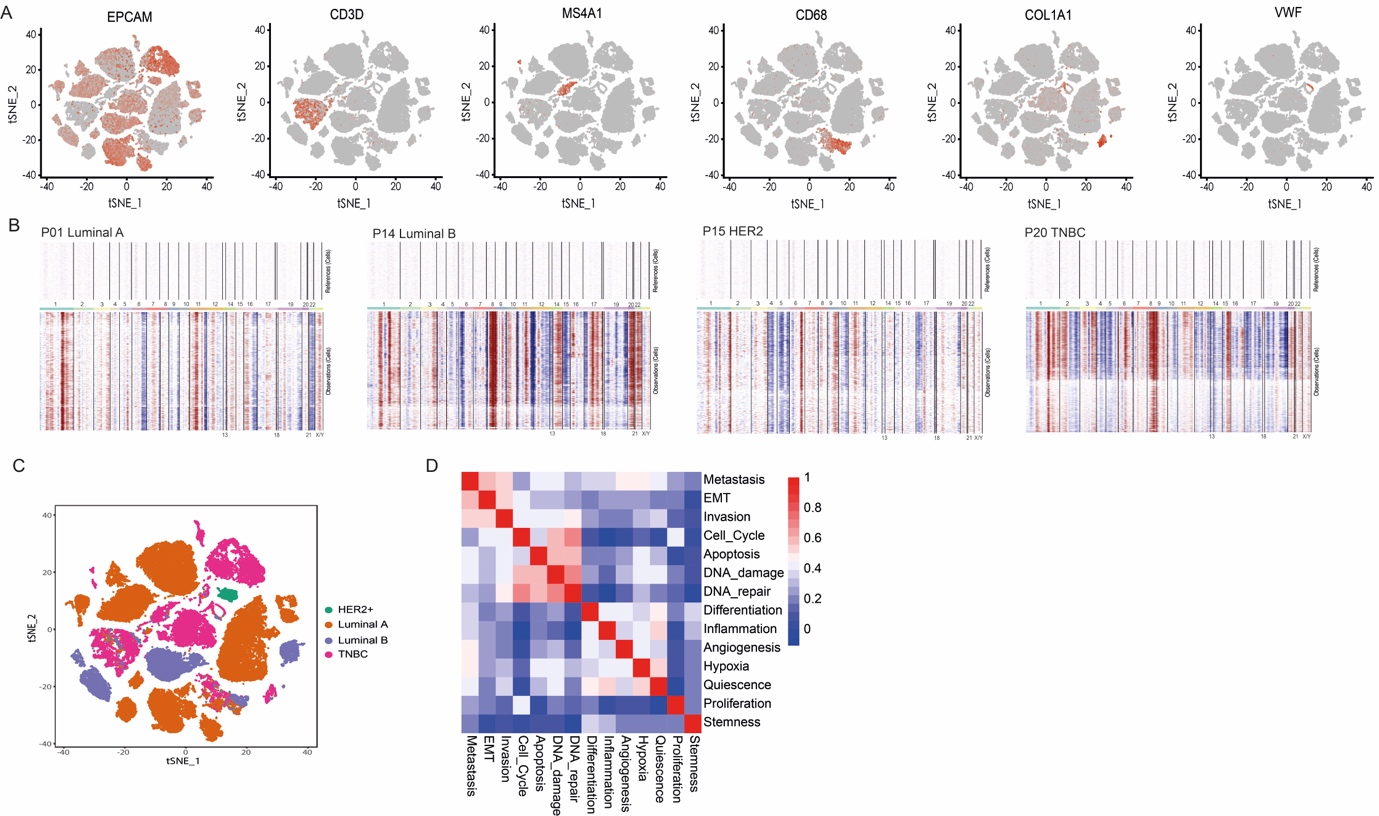
**

**Fig S1**. (A) t-SNE plots of representative genes for the annotation of cell type, including EPCAM, CD3D, MS4A1, CD68, COL1A1 and VWF. (B) Representative plots of copy number variation in P01, P14, P15 and P20. (C) t-SNE plot of the isolated malignant cells colored by molecular types. (D) Heatmap showing the correlations among these gene signatures collected by the CancerSEA database in the 17 primary tumors.

**
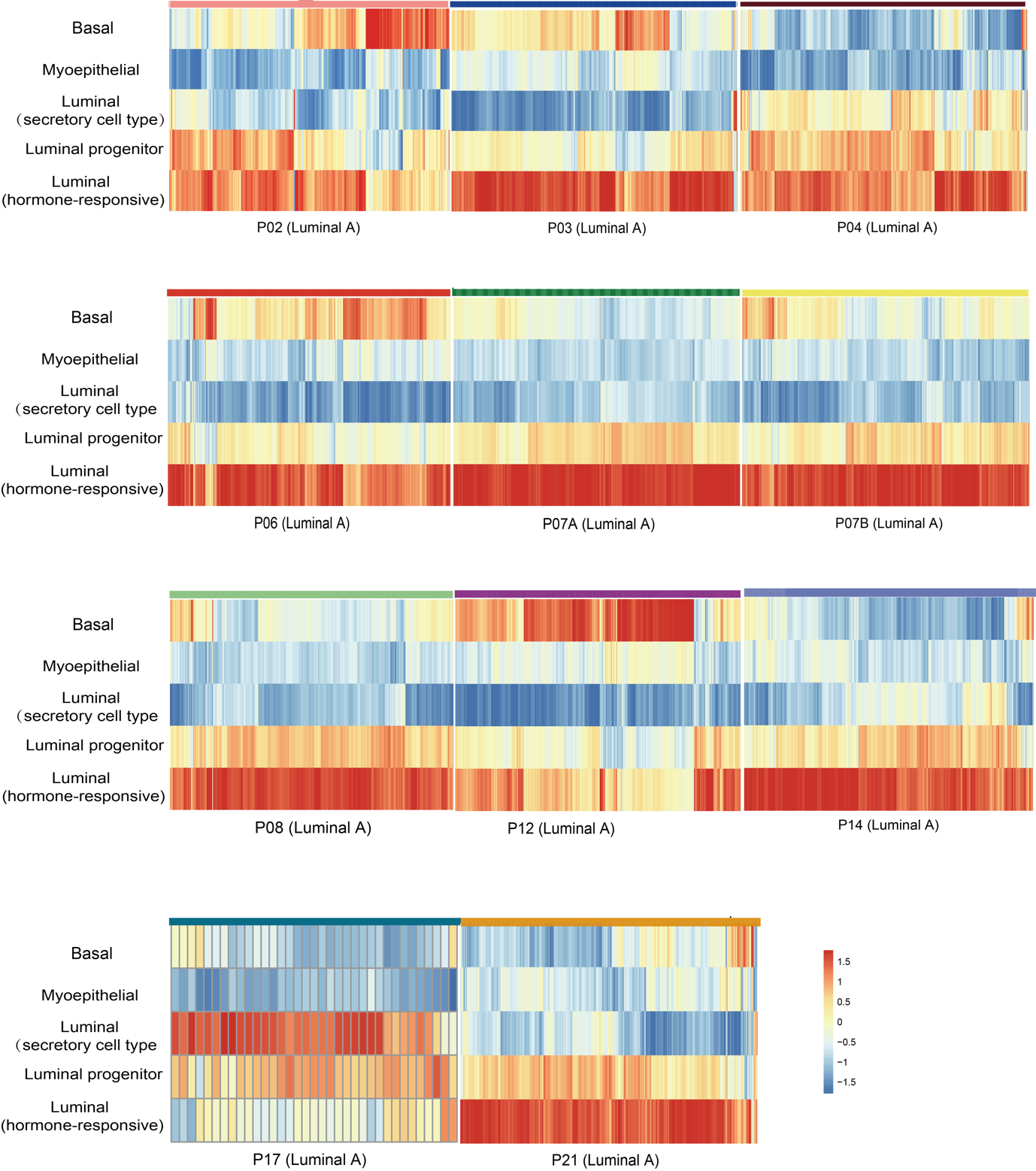
**

**Fig. S2. Heatmap of expression profiles of normal epithelial signatures in epithelial cells from 11 samples.**


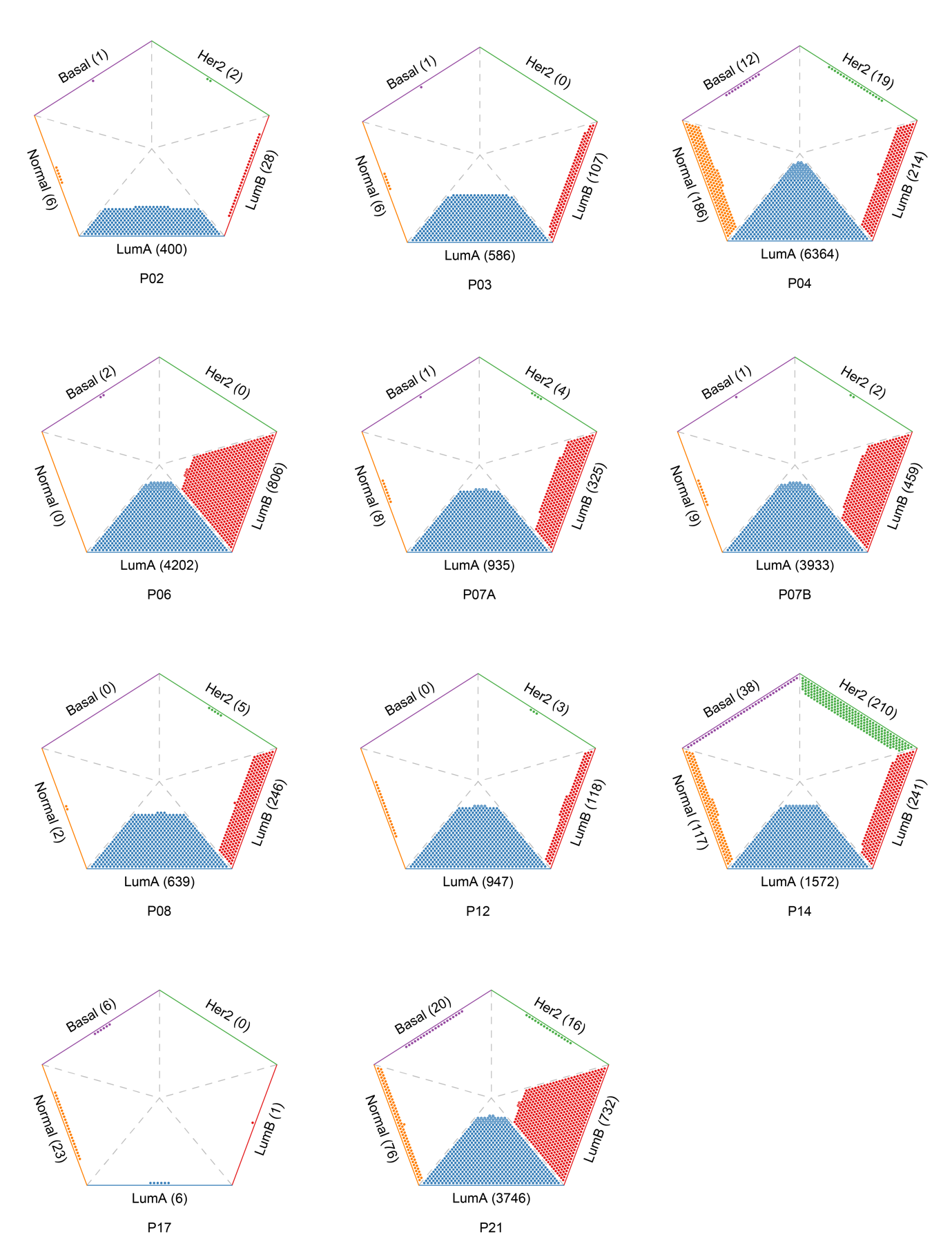


**Fig. S3. Prediction of breast cancer molecular subtype for individual tumor cell in 11 samples.**

**
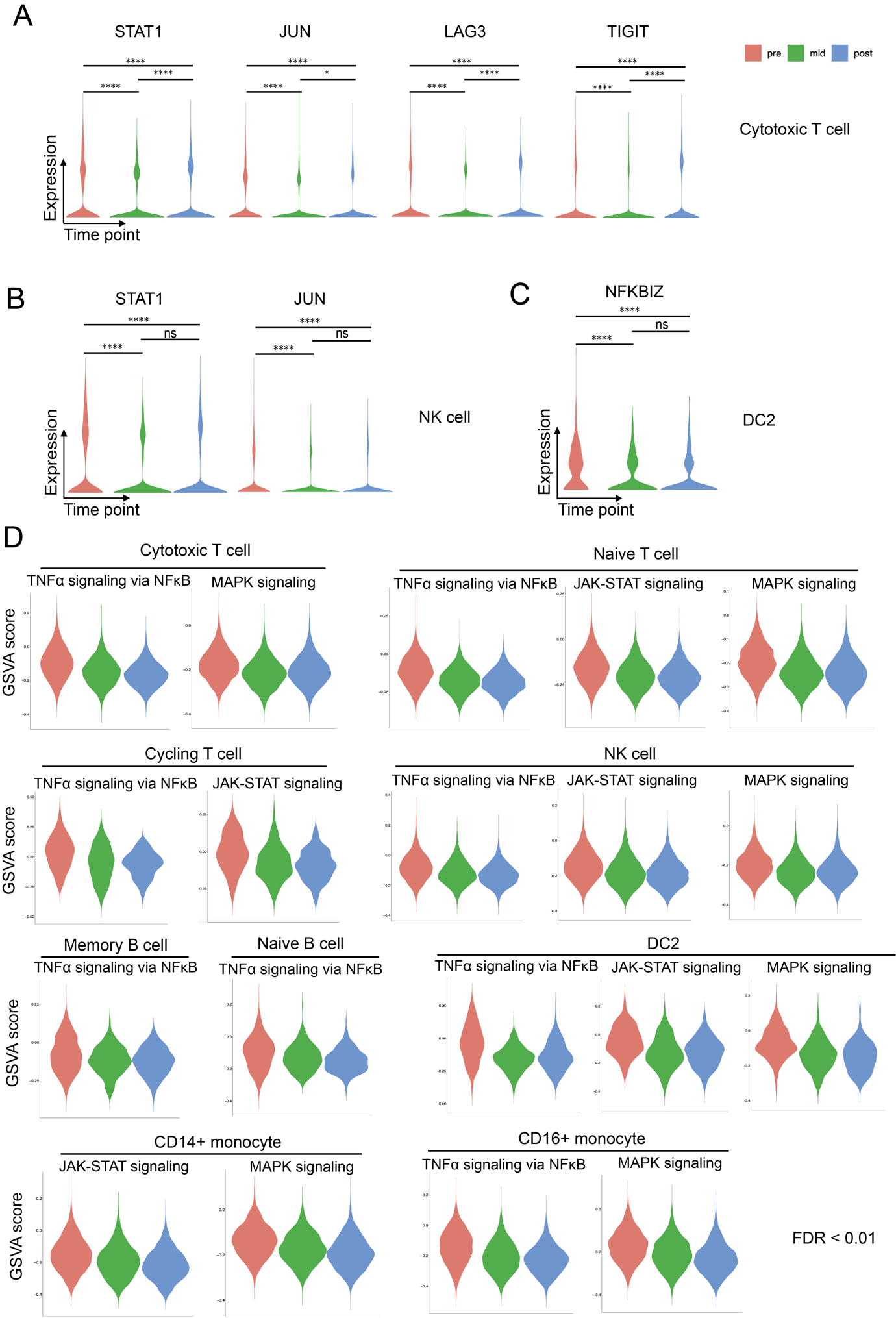
**

**Fig. S4. Differentially expressed genes and pathways identified in immune cells from PBMCs of 4 patients across 3 timepoints during NAC**. In (**A**) cytotoxic T cells, (**B**) NK cells, (**C**) DC2 cells, violin plots of differentially expressed genes. Wilcox rank sum test, * p<0.05, ** p<0.01, **** p<0.0001, ns indicates not significant. (**D**) Violin plots showing altered pathways identified by GSVA in PBMCs across different timepoints. FDR < 0.01.

1. G. X. Zheng *et al.*, Massively parallel digital transcriptional profiling of single cells. *Nat Commun* **8**, 14049 (2017).

2. A. Dobin *et al.*, STAR: ultrafast universal RNA-seq aligner. *Bioinformatics* **29**, 15-21 (2013).

3. A. P. Patel *et al.*, Single-cell RNA-seq highlights intratumoral heterogeneity in primary glioblastoma. *Science* **344**, 1396-1401 (2014).

4. Q. H. Nguyen *et al.*, Profiling human breast epithelial cells using single cell RNA sequencing identifies cell diversity. *Nat Commun* **9**, 2028 (2018).

5. D. M. Gendoo *et al.*, Genefu: an R/Bioconductor package for computation of gene expression-based signatures in breast cancer. *Bioinformatics* **32**, 1097-1099 (2016).

6. J. S. Parker *et al.*, Supervised risk predictor of breast cancer based on intrinsic subtypes. *J Clin Oncol* **27**, 1160-1167 (2009).

7. M. Efremova, M. Vento-Tormo, S. A. Teichmann, R. Vento-Tormo, CellPhoneDB: inferring cell-cell communication from combined expression of multi-subunit ligand-receptor complexes. *Nat Protoc* **15**, 1484-1506 (2020).

8. A. Butler, P. Hoffman, P. Smibert, E. Papalexi, R. Satija, Integrating single-cell transcriptomic data across different conditions, technologies, and species. *Nat Biotechnol* **36**, 411-420 (2018).

9. I. Korsunsky *et al.*, Fast, sensitive and accurate integration of single-cell data with Harmony. *Nat Methods* **16**, 1289-1296 (2019).

10. Z. Gu, R. Eils, M. Schlesner, Complex heatmaps reveal patterns and correlations in multidimensional genomic data. *Bioinformatics* **32**, 2847-2849 (2016).

11. S. Hanzelmann, R. Castelo, J. Guinney, GSVA: gene set variation analysis for microarray and RNA-seq data. *BMC Bioinformatics* **14**, 7 (2013).

12. A. Liberzon *et al.*, The Molecular Signatures Database (MSigDB) hallmark gene set collection. *Cell Syst* **1**, 417-425 (2015).

13. M. E. Ritchie *et al.*, limma powers differential expression analyses for RNA-sequencing and microarray studies. *Nucleic Acids Res* **43**, e47 (2015).

14. X. Guo *et al.*, CNSA: a data repository for archiving omics data. *Database (Oxford)* **2020**, (2020).

15. F. Z. Chen *et al.*, CNGBdb: China National GeneBank DataBase. *Yi Chuan* **42**, 799-809 (2020).
